# The Role of the Entorhinal Cortex in Extinction: Influences of Aging

**DOI:** 10.1155/2008/595282

**Published:** 2008-06-22

**Authors:** Lia R. M. Bevilaqua, Janine I. Rossato, Juliana S. Bonini, Jociane C. Myskiw, Julia R. Clarke, Siomara Monteiro, Ramón H. Lima, Jorge H. Medina, Martín Cammarota, Iván Izquierdo

**Affiliations:** ^1^Centro de Memória, Instituto de Pesquisas Biomédicas, Pontifícia Universidade Católica do Rio Grande do Sul, Avenue Ipiranga 6690, 2nd floor, 90610-000 Porto Alegre, RS, Brazil; ^2^Centro Universitário IPA, Rua Cel. Joaquim Pedro Salgado 80, 90420-060 Porto Alegre, RS, Brazil; ^3^Departamento de Fisiologia, Facultad de Medicina, Universidad de Buenos Aires, Paraguay 2155, 7th floor, 1121 Buenos Aires, Argentina

## Abstract

The entorhinal cortex is perhaps the area of the brain in which neurofibrillary tangles and amyloid plaques are first detectable in old age with or without mild cognitive impairment, and very particularly in Alzheimer's disease. It plays a key role in memory formation, retrieval, and extinction, as part of circuits that include the hippocampus, the amygdaloid nucleus, and several regions of the neocortex, in particular of the prefrontal cortex. Lesions or biochemical impairments of the entorhinal cortex hinder extinction. Microinfusion experiments have shown that glutamate NMDA receptors, calcium and calmodulin-dependent protein kinase II, and protein synthesis in the entorhinal cortex are involved in and required for extinction. Aging also hinders extinction; it is possible that its effect may be in part mediated by the entorhinal cortex.

## 1. INTRODUCTION

Since extinction is perceived as the waning of a CR, it might be taken for the expression of forgetting [1]. However, real forgetting involves the actual disappearance of memories. Instead, contrarily to this, extinguished responses usually recover spontaneously with the passage of time [2]; in addition, upon retraining, they recover very rapidly [3–5]. This is usually taken to indicate that extinguished memories do not disappear but are just made less available for retrieval (Milad, 2006). Therefore, the most widely accepted view is that extinction is just one more form of learning, in which the CS is dissociated from the former unconditioned stimulus (US) and reassociated with a new US which consists precisely in the absence of the former US [2]. In other words, a new CS-no US association is formed which supersedes the former CS-US association [2, 6], and a new conditioned response (CR) develops, usually the omission of a formerly learned response. Gale et al. (2004) have in fact produced evidence that the basolateral amygdala stores fear memories for a rat's lifetime.

Some recent evidence, however, raises the alternative possibility that extinction may involve the actual erasure of a memory trace. Extinction can be made more intense by increasing the time that rats are exposed to the absence of a footshock US in an inhibitory avoidance conditioning paradigm. In these conditions, spontaneous recovery may not be (readily) seen, and the reinstallment of the original avoidance response requires again gene expression and protein synthesis in the hippocampus [6], as it would be if it were a new response [7]. Very importantly, two different groups [8, 9] have found that if conditioned startle (a mild form of conditioned fear) is extinguished 10 to 60 minutes or less after the original training, there is no spontaneous recovery; if, instead, extinction is carried out 24 or 72 hours after training, there is rapid reinstatement of the original response. Mao et al. also observed that intra-amygdala D-cycloserine administration not only enhanced extinction, but also, in addition, reversed a GluR1 increase caused by the original training. The findings of Myers et al. and Mao et al. might be important for therapeutic purposes. There has been a search for drugs or treatments that may effectively erase a fearsome memory. Most findings have been negative. However, some reports indicate that two of these treatments, the *β*-adrenergic antagonist, propranolol (see [10]), and the gaseous anesthetic sevofluorane [11] can produce selective amnesia for emotional memories if administered at the time of encoding, but usually not later. The pretest administration of *β* blockers into entorhinal cortex, hippocampus, amygdala, or anterior cingulate cortex hinders retrieval (Barros et al., 2000). Retrieval often triggers a reconsolidation of learned responses [12]. Postretrieval propranolol hinders further recollection of traumatic memories in humans [13]. The potential usefulness of this finding is obvious. As said, many forms of learning that cannot be readily qualified as CRs can also be extinguished, and this is in fact why it is widely used in the psychotherapy of learned fear. It was originally advocated for the treatment of phobias by Freud in 1920s, but he gave it another name (habituation), which is a different form of learning [3, 14] (see below). Extinction has been also given other names when used for psychotherapeutic purposes [15], such as exposure therapy [16] or flooding [17, 18]. But it consists in all cases of what Pavlov [3], Konorski [4], and Rescorla [2] called extinction [19, 20].

## 2. EXTINCTION IS NOT HABITUATION

There are similarities and differences between extinction and habituation. As defined by Pavlov [3] and by hundreds of others after him [4, 14, 21], habituation consists in the gradual reduction of the natural, unlearned response to an unassociated stimulus or constellation of stimuli; that is, of the response to novelty. “As we sit by a highway we often quickly come to ignore the sounds of passing auto-mobiles” [21]. The same can be said of exploration of a new environment [14], of smelling the same odor for a long time, and so forth. The response to novelty or to a novel stimulus or set of stimuli is remarkably similar across species and stimuli, and involves arousal and movements of the eyes, ears, head, or body toward the source of the stimuli; it is called the “orienting” [22] or “what is it?” reflex [3]. The hippocampal molecular correlates of the response to a novel stimulus have been studied only recently. It involves the activation of different protein kinases in the hippocampus [23–25], and by the phosphorylation of the constitutive transcription factor CREB (cAMP response element binding protein) [26, 27]. This sequence of events underlies the effects of novelty on the formation of long-term memories, as part of a process of behavioral tagging [28]. Like extinction, habituation results from the repetition of a stimulus; but of a novel stimulus rather than one that had been used to form a previous association [3]. However, unlike extinction [2, 6], habituation is widely viewed as nonassociative. Also unlike extinction, habituation must be differentiated from fatigue. Pavlov [3] considered both habituation and extinction as forms of “internal inhibition”, as opposed to the stimuli that cause distraction and eventually may induce dishabituation, which he and his followers considered as examples of “external inhibition”. A major difference between both forms of “inhibition” is that whereas the “internal” type leads to diminished arousal levels, and even eventually to sleep, external inhibition causes an enhancement of arousal or alertness [3, 4]. Dishabituation has been more recently viewed as another form of learning [29], separate from [30] or linked to sensitization [31].

## 3. EXTINCTION IS NOT FORGETTING

Real forgetting involves the actual erasure of learned information. It may rely on the atrophy and eventual disappearance of synapses by disuse, as described by Eccles (1955). Indeed, we forget the face of people we saw just once or twice and then never again, unless they were highly arousing or emotionally important [32, 33]. Memories are believed to be formed and stored in synapses since Cajal [34] (see [33, 35]). In contrast, extinguished responses, knowledge, or behavior are reinstated immediately after a presentation of the US even if unassociated with the cue [36]. Relearning after extinction is usually much quicker than the original learning [5]. In addition, it may occur even if the US is presented out of context; without pairing with the CS or even outside of the training apparatus (i.e., the so-called “reminder foot-shock”, [37]). As commented above, it might be possible to proceed to the formal disappearance of a memory through an extinction procedure [6, 8, 9], see; [19]. Further indirect evidence for this is suggested by Lin et al. (2003a,b) who reported that, in the amygdala, the phosphatase calcineurin may weaken memory traces originally built by phosphatidylinositol 3-kinase mediated phosphorylation, and thereby generate extinction. In addition, recent results indicate that extinction reverses conditioning-induced enhancement of surface expression of AMPA receptor subunits in synaptsomes prepared from the lateral amygdala [38]. However, in none of the experiments which suggest that extinction may involve trace erasure is there sufficient evidence to go beyond the demonstration of a formal erasure, not of a real and incontrovertible erasure of a trace. There might always be a fragment or a hidden or otherwise strongly inhibited (repressed?) component of the memory trace thought to be lost somewhere in the brain, and it may reappear unexpectedly long after it was thought to be forgotten. One must bear in mind that the most widely used forms of psychotherapy are based precisely on this premise (see [39]).

## 4. BRAIN CIRCUITS IN EXTINCTION

Several fMRI studies show an activation of prefrontal areas, notably the ventromedial prefrontal cortex (vmPFC), together with reduced blood flow in the basolateral amygdala (BLA) [40–42] and/or the hippocampus (Milad et al., 2006); [43], in the extinction of conditioned fear responses. Importantly, the data fit with the previous evidence for a crucial role for the vmPFC ([44–47], but see also [48, 49]), and with important roles for the BLA [50, 51] and the hippocampus in retrieval and in extinction [52, 53]. Circuits linking the vmPFC with the amygdala [40] and the hippocampus (Sotres-Bayon et al., 2007), [42] in extinction have been proposed. A separate role for each of these two pathways in extinction has been envisaged by Corcoran and Quirk [54]. Circuits linking the vmPFC and as well as the dorsolateral PFC with the hippocampus have also been recently described for memory consolidation [55]; the vmPFC-hippocampus link has actually been viewed as obligatory both for consolidation and reconsolidation [41]. In more than one respect, the physiology of extinction learning is similar to that of the noninhibitory, or “regular” forms of learning; that is, the acquisition and storage of the “original” tasks that are later to be extinguished [56]. This of course agrees with the now widely accepted notion that extinction is just one more form of learning [2]. Localized brain microinfusion studies have shown that, depending on the task, the hippocampus [36, 52], the BLA [50, 51], the vmPFC (in conditioned taste aversion), and the insular cortex [56] are involved in, and are necessary for, extinction. The sequence of molecular events involved most of these regions includes glutamate N-methyl-D-aspartate (NMDA) receptors, protein kinase A, and protein synthesis in all areas studied (Vianna et al., 2004), calcium and calmodulin-dependent protein kinase II (CaMKII) in some [52, 57], and the extracellular signal regulated kinases (ERKs) in others [51]. Overall, these molecular requirements are analogous to those of memory consolidation of the original tasks [33], which further stresses the point that extinction is indeed a form of learning. In all cases, the molecular findings on extinction were determined by the use of receptor antagonists (AP5), inhibitors of CaMKII (KN62 or KN93), PKA inhibitors (Rp-cAMPs, KT5720 or others), ERK1/2 inhibitors, and protein synthesis inhibitors or inhibitors of gene expression. In the case of NMDA receptors, the partial allosteric agonist D-cycloserine has also been studied (see below). Even though all brain areas that participate in extinction in one or other task have been found to use signaling pathways and involve protein synthesis (see above), the signaling of gene expression by protein kinase cascades must surely be different across tasks and across brain areas [57–59]. For example, the ERKs are involved in extinction of auditory fear conditioning in amygdala [60] and in the extinction of contextual fear conditioning [59] and of inhibitory avoidance in hippocampus [52] but not in entorhinal cortex [57]. The extinction of conditioned taste aversion has different molecular requirements in the insula [56] and the amygdala (Bahar et al., 2003), and both are different to those reported in amygdala, hippocampus, entorhinal, or medial prefrontal cortex in other tasks (see above).

## 5. THE ENTORHINAL CORTEX: A ROLE IN LEARNING AND A ROLE IN EXTINCTION

Several early studies using localized brain lesion or stimulation techniques [61–64] and one recent pharmacological study [57] point to a crucial role of the entorhinal cortex (EC) in extinction, mostly of aversive tasks. Lesions of the entorhinal cortex inhibit not only various forms of extinction in rats but also some forms of habituation [65, 66]. Indeed, the best and most illustrative source of evidence in favor of a fundamental role of the entorhinal cortex in extinction, and indeed in all forms of learning, is human pathology: from the analysis of the famous amnestic patient H.M. [67, 68] to that of humans with mild cognitive impairment and/or with early Alzheimer's disease ([69], see below). A few studies of lesions of the EC in animals have failed to produce any result on extinction [70–72]. But some of these negative studies have also failed to detect influences of EC lesions on acquisition and retention [70] and simple discrimination [72], which disagrees with the vast majority of papers on the role of the EC in learning (see above, and [57, 69, 73] for references). In several of the negative results with entorhinal lesions, these were incomplete or encompassed other areas as well. Both the lesion and the stimulation techniques that were in vogue 20 or more years ago often gave artifactual results attributable to spread to neighboring physiologically unrelated areas [74]. In many cases, those results have not been confirmed by the more selective and circumscribed imaging, histo- or neurochemical results of the last decade or so. No doubt the entorhinal cortex must be a key component of any circuit that includes the vmPFC, the BLA, and the hippocampus, particularly one that links the former to the latter two, as has been suggested for extinction [54] (see above). First, a very large number of afferent and efferent connections between the vmPFC and the hippocampus and amygdala relay in the entorhinal cortex [75]. Second, the entorhinal cortex is the afferent and efferent relay for BLA and hippocampal connections with other regions of the rest of the cerebral cortex, all of which are connected to the entorhinal cortex [76]. Van Hoesen [76] has in fact stated that “it is clear that the entorhinal cortices receive potentially a significant portion of the sensory output generated by forebrain structures and this includes both interoceptive and exteroceptive information. In structural terms, it could be argued that the entorhinal cortex would be privy to or receive a digest of nearly all neural reactions and many of the combinations or permutations that may result. Third, the entorhinal cortex probably plays an active learning role rather than a role as a mere relay in extinction, as microneuropharmacological studies suggest. Fourth, and perhaps very importantly, medial EC neurons display positional firing properties that are somewhat different from, but related to, that of hippocampal place cells [77, 78]. Importantly, short-term memory lasting up to 3 or 4 hours is known to be processed mainly by the entorhinal cortex [23, 24, 33] and does not have extinction (Cammarota et al., 2006). Short-term memory is in charge of cognitive processing while long-term memory is being slowly built-up (Izquierdo et al., 1978), [33]. By the nature of its function, it should not have extinction (Izquierdo et al., 1978), [53], and indeed it does not leave biochemically identifiable traces [23, 33].

## 6. MOLECULAR BASIS OF THE ROLE OF THE ENTORHINAL CORTEX IN EXTINCTION

The molecular basis of inhibitory avoidance [33] and other forms of learning [58] has been studied in detail in recent years. In the case of extinction, it was studied in the ventromedial prefrontal cortex (vmPFC), the basolateral amygdala (BLA), the CA1 region of the hippocampus, the insular cortex (for conditioned taste aversion), and in the entorhinal cortex. The area of the brain in which the biological basis of extinction has been studied in most tasks is the vmPFC (see above). This area connects to the BLA and the hippocampus in order to regulate extinction, and this connection is through the entorhinal cortex [75, 76]. The dorsal hippocampus has been studied in relation to extinction very extensively, but almost exclusively in one trial step-down inhibitory avoidance [36], (Vianna et al., 2004), [6, 52, 79]. This is the task in which the molecular basis of consolidation is best known [33]. Extinction of this form of learning is indeed susceptible to the deleterious effect of the glutamate NMDA receptor blocker, 2-amino-5-phosphono pentanoate (AP5), the CaMKII inhibitor, KN93, and the protein synthesis inhibitor, anisomycin, infused into the entorhinal cortex at the time of the first of a series of retrieval sessions [57]. NMDA receptors, CaMKII, and protein synthesis are crucial for the formation of a new memory and, of course, for consolidation of this task in the hippocampus [33]. Therefore, both lesion and microinfusion experiments support a role for the entorhinal cortex in extinction; which was predictable from anatomical knowledge [76].

## 7. AGING AND EXTINCTION

It is widely agreed that aging is accompanied by a cognitive decline both in laboratory animals and in humans. Behavioral and molecular aspects of this decline have been studied extensively in the last two decades (see [80]). Recent studies have specifically demonstrated a decline of the capacity to extinguish in aged rats [81–84]. Perhaps the first to study this systematically in laboratory animals was Schneider-Rivas and his group. The decline of extinction seen in old rats correlates with changes in brain serotonin and 5-hydroxy-indole acetic acid in neocortex, hippocampus, thalamus, and dorsal raphe nucleus compatible with predictions from the serotonin hypothesis of depression, as well as with other brain neurochemical correlates ([82], see also [84, 85]). Others have reported an abnormality of forced extinction in aged rats submitted to removal of the escape platform in a water maze ([83, 86–88], see also [89]). In aged rats, this procedure quickly leads to immobility, which the authors have termed “despair“ behavior by analogy with “learned helplessness” paradigms, and which they view as a model of depression [87, 88]. The immobility is accompanied by a number of symptoms of anxiety, and by a large number of neurotransmitter changes both in striatum and in hippocampus [86, 88]. The immobility triggered by forced extinction in aged rats can be reduced by chronic desimipramine, but is actually enhanced by chronic fluoxetine, however [87]. In the forced extinction experiments in the water maze, the animals find themselves all of a sudden without the regular escape that they had learned to attain, which surely is traumatic and should cause despair. Forced extinction might happen as a result of the losses suffered by the aged, which have been so often cited as triggers of depressive episodes. When the aging person loses friends or family, or is forced to retire, or finds to have lost sensory, mental or physical powers, (s)he automatically suffers the forced extinction of a rich and large variety of responses. The cues are there: objects, pictures, remembrances, smells, sounds pertaining to the elements lost; but the response is prevented from happening because the elements themselves are gone forever. This usually occurs with pain and often with despair; and may be viewed as a nonadaptive form of extinction. The picture can be very distressing and thus lead the way to posttraumatic stress [16, 20]. The deficit of extinction in aging reported by most authors may have serious consequences, such as a proneness to perform dangerous behaviors and therefore be exposed to genuine fear situations (Izquierdo et al., 2004), ([90], see [47]).

## 8. AGING AND THE ENTORHINAL CORTEX

Perhaps the region of the brain which ages more rapidly is the entorhinal cortex. Normal aging has been known for many years to be accompanied by a reduction of neuron and synapse counts in many regions of the cerebral cortex, particularly the entorhinal cortex and then the hippocampus. The earliest occurrence of prototypical lesions in Alzheimer's disease is usually considered to be in the entorhinal cortex ([69], Jellinger et al., 1991, see [91, 92]). However, in a sizable proportion of normal aged persons lesions typical of Alzheimer's disease such as neurofibrillary tangles and neuritic plaques are also seen (Jellinger et al., 1991), [93–96], together, of course, with computerized tomography or other imaging changes suggestive of a degree of brain atrophy [91, 96, 97]. The question has been asked whether the mild cognitive impairment often seen in the aged correlates with a larger number of such lesions than that seen in the normal aged subjects, and/or with a peculiar concentration of them in the hippocampus and entorhinal cortex [96, 98]. Recent findings indicate that the answer to both questions is positive [99, 100]. Years ago it was suggested that the early atrophy of the hippocampus and particularly the entorhinal cortex could be an early marker of Alzheimer's disease [91, 92]. This correlates with the high incidence of lesions viewed as typical of Alzheimer's in the entorhinal cortex and in the hippocampus, in that order of importance in that disease [69], (Jellinger et al., 1991). Recent observations are somewhat more cautious [100, 101] and suggest that the combined use of imaging techniques plus that of cerebrospinal fluid biomarkers [101] are more likely to yield an adequate monitoring of the preclinical diagnosis of Alzheimer's in as much as the occurrence of lesions and of imaging changes in the normal and the demented elderly overlaps perhaps more than was originally thought [96, 101]. However, many studies indicate that cortical losses in the hippocampus and entorhinal cortex of elderly patients do predict mild cognitive impairment [102]. This prediction is more consistent than that of the eventual conversion of mild cognitive impairment into full-fledged Alzheimer symptomatology [103]. The dissociation of hippocampal and entorhinal memory functions has been difficult and fraught with pitfalls in as much as there is such a close interconnection between the two [75] and lesions of both structures cause very large and complete memory losses in animals (see [73]). There has been one purportedly successful attempt to dissociate the contribution of hippocampus and entorhinal cortex to different aspects of memory function in the elderly; namely, conscious recollection and familiarity-based judgments [104]; but this should be complemented by observations on other forms of memory. To be sure, aging-related changes have not only been described in the entorhinal cortex and the hippocampus, but also in other regions of the brain involved in learning and in extinction. In a very careful study, Burgmans et al. [105] reported that prefrontal cortex atrophy (particularly of the orbital region) is seen in elderly patients with cognitive impairment and more intensely in those with dementia, and is a better predictor of the latter over a 6-year period than medial temporal lobe atrophy. In the rat basolateral amygdala, a hypertrophy of the dendritic tree independent of sex was seen in aged (20–24-month old) animals as compared with 3–5-month old animals (Rubinow et al., 2007). How does this hypertrophy relate to the atrophy described in the prefrontal cortex and in the medial temporal lobe de-scribed in aging mammals, including humans, it is hard to tell.

## 9. CONCLUSIONS

The entorhinal cortex plays a key role in cognition. It contributes to, and processes, information that the rest of the cortex, particularly the prefrontal areas, sends to it in order to be relayed to the hippocampus and amygdala, as part of the acquisition, retrieval, or extinction of many forms of learning. In addition, the entorhinal cortex also processes information generated by the hippocampus and sends it to the neocortex, and interconnects the hippocampus with its main regulatory nucleus complex, the amygdala. Thus the entorhinal cortex is crucially involved in all aspects of learning. Its role in extinction has been best studied in aversive tasks. The entorhinal cortex on one hand and extinction on the other suffer severe losses with aging. The changes are more marked in humans with mild cognitive impairment, and much worse in Alzheimer's disease, in which the entorhinal losses are diagnostic at early stages. The impairment of extinction seen in old age may be related in part to entorhinal cortex damage.
